# Penile enlargement with methacrylate injection: is it safe?

**DOI:** 10.1590/S1516-31802013000100009

**Published:** 2013-02-01

**Authors:** Fabio Cesar Miranda Torricelli, Enrico Martins de Andrade, Giovanni Scala Marchini, Roberto Iglesias Lopes, Joaquim Francisco Almeida Claro, Jose Cury, Miguel Srougi

**Affiliations:** I MD. Urological Surgeon, Division of Urology, Faculdade de Medicina da Universidade de São Paulo (FMUSP), São Paulo, Brazil.; II MD. Head of the Sexual Medicine Group, Division of Urology, Faculdade de Medicina da Universidade de São Paulo (FMUSP), São Paulo, Brazil.; III PhD. Professor and Chairman of the Division of Urology, Faculdade de Medicina da Universidade de São Paulo (FMUSP), São Paulo, Brazil.

**Keywords:** Penile implantation, Polymethyl methacrylate, Penis, Erectile dysfunction, Penile diseases, Implante peniano, Polimetil metacrilato, Pênis, Disfunção erétil, Doenças do pênis

## Abstract

**CONTEXT:**

Penis size is a great concern for men in many cultures. Despite the great variety of methods for penile augmentation, none has gained unanimous acceptance among experts in the field. However, in this era of minimally invasive procedure, injection therapy for penile augmentation has become more popular. Here we report a case of methacrylate injection in the penis that evolved with penile deformity and sexual dysfunction. This work also reviews the investigation and management of this pathological condition.

**CASE REPORT:**

A 36-year-old male sought medical care with a complaint of penile deformity and sexual dysfunction after methacrylate injection. The treatment administered was surgical removal. Satisfactory cosmetic and functional results were reached after two months.

**CONCLUSIONS:**

There is a need for better structured scientific research to evaluate the outcomes and complication rates from all penile augmentation procedures.

## RESUMO

**CONTEXTO:**

O tamanho peniano é uma grande preocupação para homens em diversas culturas. Apesar da grande variedade de possíveis métodos para o aumento peniano, nenhum ganhou aceitação unânime por especialistas no assunto. Mas na era dos procedimentos minimamente invasivos, a terapia de injeção para aumento peniano tem se tornado mais popular. Aqui relatamos um caso de injeção de metacrilato no pênis que evoluiu com deformidade peniana e disfunção sexual. Este trabalho também revisa a investigação e gerenciamento desta afecção.

**RELATO DE CASO:**

Um homem de 36 anos procurou atendimento médico com queixa de deformidade peniana e disfunção sexual após injeção de metacrilato. Remoção cirúrgica foi realizada como tratamento. Após dois meses, satisfatórios resultados cosmético e funcional foram alcançados.

**CONCLUSÃO:**

Trabalhos científicos mais bem estruturados para avaliar as taxas de complicação e resultados de todos os procedimentos para aumento peniano são necessários.

## INTRODUCTION

Penis size is a great concern for men in many cultures, based on the belief that “bigger is better” and the idea that the penis is central to men’s virility. Today, men often feel a need to enlarge their penises in order either to improve their self-esteem or to satisfy and impress their partners. The demand for penile augmentation continues to increase, although cosmetic surgery to enlarge the penis remains highly controversial and surgical outcomes are still uncertain.

The vast majority of men who request penile enhancement surgery usually have a normally functioning penis. The current main procedures for augmentation phalloplasty are penile lengthening and girth enhancement by means of dermofat graft. However, several methods for increasing penis size have been described in the literature, such as abdomino/pubopelvic liposuction, suspensory ligament dissection, skin flaps and different kinds of injections.^1^

Despite the variety of methods available, none has gained unanimous acceptance among experts in the field. However, in this era of minimally invasive procedures, injection therapy for penile augmentation has become more popular and it has been proposed by physicians as the easiest way to obtain a bigger penis. Paraffin, mineral oil, metallic mercury, petroleum jelly, transmission fluid, subcutaneous stone implantation and autologous fat implantation are some of the various materials that have been tried,^2^^,^^3^^,^^4^^,^^5^ although liquid fluid silicone is the only one approved by the United States Food and Drug Administration (FDA) for some cosmetic procedures.^6^ None of them has been tested and approved for penile enlargement. In this paper, we present a case of methacrylate injection in the penis that had been oriented by a medical team, which evolved with penile deformity and sexual dysfunction. We also review the investigation and management of this pathology.

## CASE REPORT

**Figure 1.**
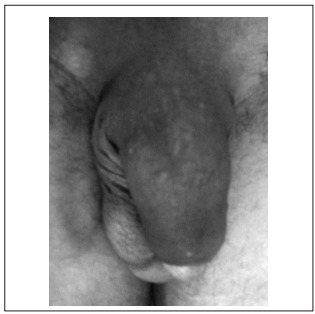
Penis appearance after methacrylate injection.

A 36-year-old male presented with an irregular hard mass along the whole length of the penile shaft. It was bigger near to the pubis in the ventral portion of the penis (Figure 1). The patient also complained of difficulty in obtaining a hard enough erection for satisfactory intromission, and pain was usually present during attempts at sexual intercourse. Despite the great dimension of the mass, the patient did not present any voiding symptom. The glans penis was normal. The overlying skin was unaffected and there was no palpable lymph node enlargement.

The patient noted this penile deformity after injection of methacrylate in a private clinic two years earlier. According to the patient, a urologist proposed a safe and legal method for penile girth augmentation. Initially, the physician proposed three sessions to perform the complete injection, but after the first procedure, the patient noted a significant mass in the penis and refused to continue the treatment. He never became satisfied with the procedure. After two years of looking for some kind of relief for his penile condition, the patient was referred to our clinic. He was very dissatisfied with the cosmetic and functional result and wanted to recover the old penile appearance.

**Figure 2.**
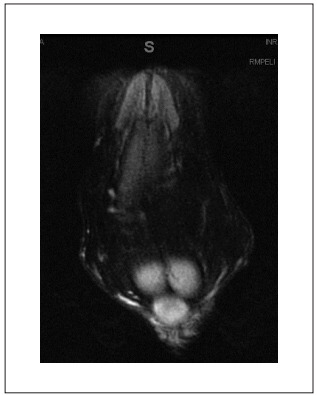
Magnetic resonance image (MRI) showing diffusely enhanced penile subcutaneous thickness and compressive effect of the mass over the corpora cavernosa and corpus spongiosum.

In our clinic, pelvic magnetic resonance imaging (MRI) (Figure 2) was performed and showed diffuse enhance of penile subcutaneous thickness, which was compatible with fibrosis, and a compressive effect from the mass over the corpora cavernosa and corpus spongiosum, mainly on the left side. This might have caused restriction of corpora cavernosa turgidity. Diffuse enhancement of Buck’s fascia thickness was also observed. The urethra was preserved. No other significant abnormalities were found in the MRI.

**Figure 3.**
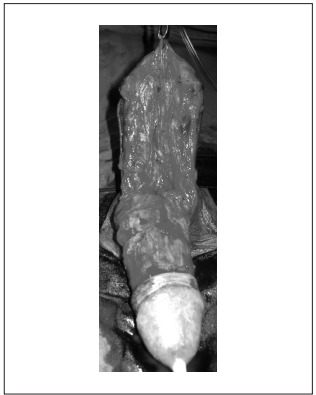
Resection of the mass and excess skin.

**Figure 4.**
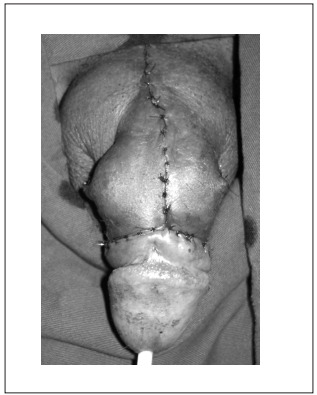
Penis final appearance after surgery.

After discussion with the patient, the medical team proposed surgical removal of the mass. The procedure was performed under spinal anesthesia in the supine position. Antibiotic prophylaxis was administered using ciprofloxacin. The patient was catheterized with Foley 16 and the access was though a circumferential subcoronal plus longitudinal incision. The mass and the excess skin were removed together (Figure 3). The neurovascular bundle was preserved. The procedure was uneventful. The penile shaft was covered with a skin flap and a Penrose drain was left in, under the skin flap (Figure 4). The surgical specimen was sent for pathological examination, which revealed that the skin presented dermal fibrosis and formation of foreign-body granuloma with amorphous material.

**Figure 5.**
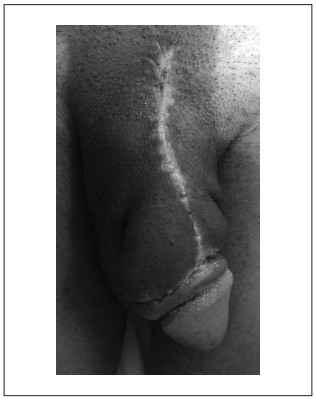
Penis appearance two months after surgery.

There were no complications during the early postoperative period. The Penrose drain was removed on the first postoperative day and the urinary catheter was left until the second day. The patient also left the hospital on the second postoperative day. No late complications were observed; the skin flap healed well, except for a small distal area; and, after two months, satisfactory cosmetic and functional outcomes were reached (Figure 5).

## DISCUSSION

Today, several interventions for penile enlargement are sold to men who desire a bigger and more attractive penis, both by physicians and by lay people, often with media support. However, the “experimental” nature of this kind of approach is almost never completely explained to these patients, who believe that they are undergoing a modern and safe procedure. The best option and outcome for penile girth augmentation remain unclear. None of the penile enhancement techniques have been approved by any of the professional societies, and the majority are performed in private settings, thereby leading to medical-legal implications and paucity of specific data.^1^ Another important issue is how to identify patients who would benefit from penile augmentation. Moreover, concerns continue with regard to deciding what the valid indications for performing such procedures would be, selecting the most suitable procedure and designating the outcome measurements.

Invasive filler injection procedures have been reported to be capable of meeting patient expectations. Yacobi et al.^7^ reported their experience with ultra-purified liquid injectable silicone for penile shaft augmentation among a considerable sample of 324 patients. Three to six sessions were performed and, after a mean follow-up of 20 months (range 1-36 months), these investigators reported that an increase in penile girth of 27% had been achieved. No complications were noted.

Many fillers are available for tissue augmentation,^2^^,^^3^^,^^4^^,^^5^ but the ideal filling substance remains unknown. Such a substance would be biocompatible, non-antigenic, non-pyrogenic, non-inflammatory, nontoxic, easy to use, stable after injection, non-migratory, natural looking and not too expensive. In our case, methacrylate injection was used for penile enhancement. Methacrylates are the salts or esters of methacrylic acid and are common monomers in polymer plastics, forming the acrylate polymers. In comparison, other synthetic fillers such as polytetrafluoroethylene and silicone, with irregular surfaces, are more prone to cause chronic granulomas reactions.^8^ Table 1 shows the lack of experience in the literature, regarding methacrylate for penile girth augmentation.

**Table 1.** Literature review on penile augmentation with methacrylate injection

Electronic databases	Search strategies	Results	
		Found	Related
PubMed	Penile girth augmentation	17	0
	Penile girth augmentation and methacrylate	0	0
SciELO	Penile girth augmentation	0	0
Lilacs	Penile girth augmentation	0	0
Cochrane Library	Penile girth augmentation	0	0

Although several options are available for penile girth augmentation, none of them is free from complications. Wassermann and Greenwald^9^ reported the case of a 42-year-old man who had had silicone injected into his corpora cavernosa 14 years prior to his presentation with edema of the penis and scrotum. The patient had palpable siliconomas obstructing the glans and required surgical resection. More recently, Silberstein et al.^6^ reported the case of a 61-year-old male hospitalized for treatment of cellulitis in his right lower extremity who, in the physical examination, was found to present a grossly edematous circumcised penis with marked firm swelling. A computed tomography scan revealed a diffusely enlarged penis, with multiple rounded structures showing peripheral calcification, of which the largest measured 2.3 cm, distortion of the soft tissues, and poor viewing of the corpus cavernosum and spongiosum. The patient admitted that a nonmedical practitioner had injected a “silicone mixture” several years earlier (the patient could only guess at around 10-15 years earlier). He was “pleased” and declined any further intervention. Shaeer et al.^10^ reported the case of a 28-year-old male who presented with a subcutaneous mass in the penile shaft, which had resulted in deformity and difficult intromission, as well as coital pain for the female partner. He had injected gel into the penis two years prior to presentation. The injected material had started to migrate and had coalesced into a painless mass. The patient requested surgical removal of the mass, which was done without complications. These cases have many points in common with the case that we presented here, thus showing that penile deformity and sexual dysfunction may be serious complications from injection of fillers for girth augmentation.

Moreover, a report on complications after injection of various polymethyl methacrylate-based dermal fillers had already been published by Salles et al.,^8^ such as tissue necrosis, granulomas, chronic inflammatory reaction and infection.

## CONCLUSION

Several fillers are available for penile girth augmentation, but the real outcomes are still uncertain and serious complications may occur. There is a need for scientific and methodological research on the outcomes and complication rates of all these procedures.
